# Eye movements as predictors of student experiences during nursing simulation learning events

**DOI:** 10.1186/s41235-025-00640-7

**Published:** 2025-07-01

**Authors:** Madison Lee Mason, Caleb Vatral, Clayton Cohn, Eduardo Davalos, Mary Ann Jessee, Gautam Biswas, Daniel T. Levin

**Affiliations:** 1 Department of Psychology and Human Development, Vanderbilt University, Nashville, USA; 2 Department of Computer Science, Tennessee State University, Nashville, USA; 3 Department of Computer Science, Vanderbilt University, Nashville, USA; 4 School of Nursing, Colorado Mountain College, Breckenridge, USA

**Keywords:** Mobile eye-tracking, Event cognition, Simulation education

## Abstract

Although the “eye-mind link” hypothesis posits that eye movements provide a direct window into cognitive processing, linking eye movements to specific cognitions in real-world settings remains challenging. This challenge may arise because gaze metrics such as fixation duration, pupil size, and saccade amplitude are often aggregated across timelines that include heterogeneous events. To address this, we tested whether aggregating gaze parameters across participant-defined events could support the hypothesis that increased focal processing, indicated by greater gaze duration and pupil diameter, and decreased scene exploration, indicated by smaller saccade amplitude, would predict effective task performance. Using head-mounted eye trackers, nursing students engaged in simulation learning and later segmented their simulation footage into meaningful events, categorizing their behaviors, task outcomes, and cognitive states at the event level. Increased fixation duration and pupil diameter predicted higher student-rated teamwork quality, while increased pupil diameter predicted judgments of effective communication. Additionally, increased saccade amplitude positively predicted students’ perceived self-efficacy. These relationships did not vary across event types, and gaze parameters did not differ significantly between the beginning, middle, and end of events. However, there was a significant increase in fixation duration during the first five seconds of an event compared to the last five seconds of the previous event, suggesting an initial encoding phase at an event boundary. In conclusion, event-level gaze parameters serve as valid indicators of focal processing and scene exploration in natural learning environments, generalizing across event types.

## Eye movements as predictors of student experiences during nursing simulation learning events

The human eye has been described as a “window into memory” (Ryan & Shen, 2020) or even a “window to the soul” (Rauthmann et al., 2012). These claims stem from foundational theories in cognitive psychology, such as the “eye-mind link” (Just & Carpenter, 1980; Reichle et al., 2012), which broadly posit that eye movements reflect cognitive processing. This framework suggests that ocular dynamics such as fixations, saccades, and pupil diameter, are controlled by a combination of ocular constraints and cognitive processes such as attention, scene searching, memory encoding, and decision-making (Gidlöf et al., 2013). Eye movement patterns have been shown to be associated with goal-directed cognitive engagement in settings ranging from reading comprehension (Reichle et al., 2010) to scene perception (Kardan et al., 2015), to viewing instructional videos (Faber et al., 2020).

Consistent with the basic eye-mind hypothesis, basic parameters such as fixations, saccades, and pupil diameter do sometimes predict cognitions and important outcomes such as learning. Durations of fixations, where the gaze remains steady on a particular stimulus, often reveal the locus of attention and signal the processing of specific information (Shojaeizadeh et al., 2016). For example, increased fixation duration seems to consistently indicate an increased amount of cognitive load taxing an individual (Coral, 2016), and it also has been shown to predict change detection (Henderson & Hollingworth, 1999). Amplitudes of saccades, which are ballistic eye movements between fixations, appear to reflect the dynamic allocation of attention, signaling more ambient processing of information (Guo et al., 2022; Ito et al., 2017). Increased saccade amplitude has also been linked with increases in cognitive load (Coral, 2016) and decreased reading speed (Rima & Schmid, 2021). Variations in pupil diameter indicate variations in arousal and mental effort, providing insights into the cognitive engagement and attentional focus associated with the observed stimuli (Eckstein et al., 2017). For example, pupil dilation has been shown to predict memory encoding and sentence comprehension (Eckstein et al., 2017).

These gaze-cognition associations are seen within various domains of research spanning from basic cognitive processing work to social cognition, developmental psychology, and learning sciences. Studies on scene-viewing have demonstrated that fixation duration behavior is associated with the ability to flexibly update and maintain working memory (Loh et al., 2023). In the realm of social cognition, longer fixation durations on revisited items occur significantly more often during decision making tasks compared to search tasks (Gidlöf et al., 2013) and pupil dilation synchrony among team members significantly predicts team performance during a coordination task (He et al., 2021). Meanwhile, developmental work has relied on infant pupil dilation as a positive indicator of object permanence and short-term memory (Eckstein et al., 2017). Finally, in learning sciences, student eye gaze behavior while reading has been shown to predict their causal reasoning abilities about that material later on (Rajendran et al., 2018). More broadly, selective allocation of attention to task-specific information appears to be a consistent predictor of expertise across a number of domains (e.g., medicine, sports, aviation; Brams et al., 2019). This kind of research on gaze behavior has practical implications, influencing instructional design and enhancing learning experiences (Wright et al., 2022; Xie et al., 2021). These interdisciplinary findings collectively underscore the pivotal role of gaze behavior in cognition, and consequently performance and task outcomes, and illustrate the understandable desire to develop a unified gaze metric model to make sense of overlapping but yet domain-specific findings (Reichle et al., 2012).

## Gaze-to-cognition associations are context dependent

Although substantial research has highlighted the evident connection between gaze behavior and cognitive states, it is important to acknowledge the nuanced nature of these relationships, which can give rise to contrasting findings across studies. Variability in individual’s use of cognitive strategies, task demands, and contextual factors contribute to divergent gaze pattern associations with cognitive states (Faber et al., 2020). Faber and colleagues (2020) found distinct gaze-cognition links across tasks that varied in levels of required discourse processing, visual processing, and spatial allocation. For example, fixation durations were shorter during mind wandering tasks that required central focus to achieve a goal (e.g., listening to an audiobook), but fixation durations were longer during mind wandering for a task that required more extensive sampling of the visual field (e.g., reading illustrated text). Also, research has shown that fixating on a changing object both before and after it changes sometimes, but not always, induces awareness of the change (Smith et al., 2012). Finally, it is well known that the locus of attention is not always the same as the locus of gaze (Ricciardelli & Turatto, 2011), and that fixation locations can even be the site of attentional inhibition (Mack & Rock, 1998). Thus, even relatively basic forms of attention and representation are not fully constrained by gaze, so it is important to interpret gaze with respect to contextual constraints that affect both gaze allocation and subsequent cognitive processing. Broadly, Anderson (2004) concluded that eye movements reflect cognitive processing only to the degree to which the task involved requires information encoding. In contrast, Mak and colleagues (2002) reported that eye movements reflect downstream processing after initial encoding (e.g., later retrieval processes). Recognizing these complexities makes it evident that a unified model linking gaze and cognition is not sufficient for all contexts and that further contextualization must take place to better understand when and how gaze is associated with cognition and task outcomes.

Due to the heterogeneity of gaze-performance links, it is crucial to explore these connections across a broad range of settings, both in the laboratory and in real-world environments. Doing so is particularly important if eye tracking is to be developed as a means of understanding real-world performance and learning. In classic cognitive psychology, investigations of gaze-performance associations are often limited by the methodological capabilities of eye-tracking where participants must keep still using a chin rest, responding to potentially hundreds of trials. It was not until the early 2000s that mobile eye-tracking became increasing available for research purposes (Cognolato et al., 2018), and only recently have these devices become relatively unobtrusive.

In particular, mobile eye-tracking has become useful for investigating dynamic, hands-on learning experiences. The use of simulation training is now common practice in many fields because it allows for high-fidelity learning experiences without any real-world risk (e.g., pilot training, medical education, police enforcement, and army training; Chernikova et al., 2020). Research teams have started implementing mobile eye-tracking in these simulation scenarios to evaluate the relationship among gaze, cognition, and performance, and then utilize these associations as forms of learning feedback. For example, Carroll and colleagues (2015) utilized gaze metrics, including fixation count, fixation duration, and gaze trajectories, as indicators of visual attention allocation to aid in diagnosing performance errors among tactical aviation trainees. Researchers provided the playback of trainees’ gaze footage as a form of debriefing support for an experimental group and found these trainees’ scan strategies became significantly more effective in this experimental group compared to that of the control group (Carroll et al., 2015). O’Meara et al. (2015) used a similar debriefing support system for nursing students after the students engaged in clinical simulations. Students who used the eye-tracking-enhanced debriefing system demonstrated significantly greater situational awareness in a follow-up simulation performance. These studies and others (e.g., Rudi et al., 2020) have made excellent use of eye tracking technology to provide attentional feedback to students, but less work has explored more fundamental relationships between basic gaze parameters, cognition, and performance in these particular educational settings.

Key outcomes in nursing education include task achievement, teamwork quality, communication effectiveness, and self-efficacy (Jeffries, 2021). These outcomes are closely linked to cognitive states such as focus. Novice nurses tend to utilize increased internal focus—where attention is directed inward toward one’s own thoughts and actions—more than experienced nurses. This is believed to reflect a reliance on analytical reasoning rather than intuition (Benner, 2004; Tanner, 2006). Intuition is described as an automatic reasoning pattern that is developed and strengthen through clinical experience (Manetti, 2019). This analytical approach is necessary for building foundational skills, as novices lack the experience required for intuitive decision-making (Benner, 2004). Based on gaze-cognition links discussed earlier, one can imagine the implications of increased internal focus and analytical reasoning on gaze behavior and on task performance. For example, these students could be producing more inefficient gaze behaviors such as increased scene exploration (i.e., saccade amplitude) when it is not necessary to do so. Further, novice students may have an increased cognitive load that is reflected in changes in pupil diameter. Consequently, these novice students may suffer from reduced task achievement and self-efficacy.

Given the important role of context in developing gaze-performance links, one major challenge with interpreting real-world gaze data is the difficulty in specifying these contexts. One approach is to follow the lead of Faber et al (2020) and detail how specific tasks may influence the relationship between gaze, cognition, and performance. However, it is also possible to focus in more detail within tasks and stimulus materials and assess the degree to which specific events may influence links between gaze, cognition, and task outcomes. Consequently, one possible analytical approach can be derived from theories that specify how continuous experience is broken in the discrete events. For example, Event Segmentation Theory explains how individuals segment their continuous perceptual experience into meaningful event units by default (Zacks et al., 2007). Event Segmentation Theory (Kurby & Zacks, 2008) posits that we engage in segmentation in a hierarchical manner such that our perceptual experience is organized into coarse events (i.e., caring for a patient) and also fine events (i.e., greeting the patient, performing a physical exam, and giving the patient medication). Research has shown behavioral event segmentation (i.e., pressing a button when one meaningful event ends and another event begins) not only time-locks to human brain activity (Zacks et al., 2001) but also that the act of segmentation supports memory for events up to one month later (Flores et al., 2017). However, few studies have used events as units of analysis to investigate the relationship between eye movements, cognition, and task outcome. In one laboratory study, Eisenberg and Zacks (2016) found that during dynamic scene viewing, participants initially engage in ambient processing at the onset of a new event, as evidenced by an increase in saccade amplitude and a decrease in fixation duration. As the event unfolds, this shifts to focal processing, characterized by an increase in fixation duration and a decrease in saccade amplitude. But it is also possible to focus on between-event comparisons to test whether gaze and task outcome links can be observed when controlling for between-event differences in actions that are likely to influence gaze parameters. For example, in settings such as nursing simulation education, events vary in the prevalence of manual actions, between-person communications, and reading/writing that the participant engages in. Each of these likely influences gaze statistics, so it may be useful to control for these factors in a regression. More basic, an analysis that parses gaze statistics by events within tasks has the potential for increased statistical power in a multilevel analysis as it allows for an increased number of units of analysis, but it also requires careful modeling of random effects to avoid inflating Type I error (Barr et al., 2013).

## Current study

In this study, we tested for relationships between eye movement and judgments of task performance while participants engaged in a hands-on educational experience. We recruited nursing students to wear mobile eye-tracking glasses while they participated in clinical simulations. In the clinical simulations, students were required to attend to the needs of an interactive patient manikin by treating it based on their assessment of the patient’s vital signs. Subsequently, students reviewed and reflected on their own eye-tracking footage. As part of this review, students segmented egocentric videos (with superimposed gaze points) of their experience into meaningful event units. Students then returned to each video segment, identifying their actions and cognitive states, and judging their success in term of teamwork quality, goal achievement, communication, and self-efficacy.

Prior research on event segmentation has largely relied on third-person videos of everyday activities (e.g., Eisenberg & Zacks, 2016) or professionally edited films (e.g., Hollywood-style movie clips; Zacks, 2020). However, these studies do not reflect how individuals segment their own lived experiences in high-stakes environments such as clinical decision-making. The present study is the first, to our knowledge, in which participants segmented their own prior experience from a first-person viewpoint. This approach allows for a more personalized, context-dependent examination of event segmentation in professional learning settings, such as nursing education.

We hypothesize that event-level gaze parameters and cognitive focus would be significant predictors of event-level goal achievement, teamwork quality, communication effectiveness, and self-efficacy. In this particular study, these measures were obtained from student ratings of their own performance. Although objective performance ratings are useful as well, we focused on student self-ratings because they are integral to the simulation debriefing process.

Specifically, increased mean fixation duration is associated with focal processing and scene exploitation (Pannasch et al., 2008; Unema et al., 2005). If this form of processing reflects effective and fluid information processing, then we might expect event-level mean fixation duration to be positively associated with students’ self-reported goal achievement, team performance, communication effectiveness, and self-efficacy (König et al., 2016). Pupil diameter has been found to decrease during mind wandering (Faber et al., 2018) and to increase with cognitive processing (Zénon, 2019). Thus, we expect pupil diameter to also be positively associated with these four event-level performance judgments. Conversely, mean saccade amplitude is known to increase with active searching behavior and is positively associated with subjective effort and processing demands (Cabrall et al., 2020). In addition, prior exploratory work has found potential relationships between mean saccade amplitude and self-efficacy in nursing simulation training environments similar to those studied in this work (Vatral et al., 2023). We expect increased saccade amplitude to reflect mental effort and cognitive load resulting in a negative association with achievement, teamwork quality, communication, and self-efficacy. Additionally, in the domain of nursing, we expect increased self-reported internal focus, incorporated in our models as a covariate, to be negatively associated with these four event-level judgments of task performance.

Finally, we also explored the degree to which relationships between gaze parameters and performance judgments were homogenous within each event, across event boundaries, and across participant-defined event types. This follow-up analytical approach allowed us to determine whether specific patterns of gaze behavior were consistent within a single event type or if they shifted when participants transitioned between different events. By examining these dynamics, we aimed to understand if the nature of the task or the shift in task demands influenced the consistency of the relationship between gaze and self-reported task performance.

While our dependent measures are indeed self-reported judgments of performance rather than direct measures of actual task outcomes, they align with validated approaches used in prior research, such as Faber and colleague’s (2020) work on mind wandering, where subjective judgments are considered reliable indicators of cognitive engagement. Additionally, these judgments hold value in our application because one of the primary objectives of the nursing simulations is to foster outcomes like increased self-efficacy, making participants’ perceived performance a crucial outcome in itself. For example, self-efficacy has been shown to influence nurses’ clinical decision-making and the effectiveness of their performance (Cabrera-Aguilar et al., 2023; Lee & Ko, 2010). Student-identified markers of high and low confidence and teamwork serve as crucial guides for students to reflect on their own performance (Horcajo et al., 2022), and the possibility to detect these variations in experience via eye movements not only validates important reflective processes, but also leads to opportunities to develop teaching supports that could help automate the process of alerting students to key moments. Another advantage of exploring these variables is that they are collected as a natural part of our reflective exercise.

## Method

### Transparency and openness

In line with Transparency and Openness guidelines, this manuscript specifies how we determined our sample size, all data exclusions, all manipulations, and all measures in the study. Data, materials, and pre-registered analyses are publicly available on OSF (https://osf.io/fwvd6). Data were analyzed using RStudio, version 2023.12.0 + 369 and JASP, version 0.16.2. The research protocol for this study was approved by the Institutional Review Board (IRB) at Vanderbilt University and all procedures were conducted in accordance with the ethical guidelines and regulations outlined by the IRB.

### Participants

Pre-specialty students from Vanderbilt University’s School of Nursing were recruited to participate in this experiment during their Spring 2023 clinical simulation sessions. Prior to entering their simulation sessions, students were offered the opportunity to wear eye-tracking glasses during their simulation, and all students were asked to provide their informed consent to collect video and audio data as they performed their training activities. Sixty-five students were eye-tracked during their simulation scenario. The sample in the current study consists of 25 of those students who agreed to participate in the guided reflection and event segmentation portion of the study at a later date. Students who agreed to participate in the guided reflection were compensated with a $50 Amazon gift card.

Reflection participants’ mean age was 26.1 years old (SD = 5.3). 22 students reported their gender as female and 3 as male. Participants reported their gender identities using a drop-down menu with the following possible options: “Male”, “Female”, “Transgender male-to-female”, “Transgender female-to-male”, “Gender variant/non-conforming”, “Prefer to self-describe:”, or “Prefer not to answer”. 21 participants reported their race as “White”, 1 participant was “American Indian or Alaskan Native”, 1 participant was “Hispanic or Latino”, 1 participant was “Asian”, and 1 participant selected “Other”, reporting their race as “Middle Eastern, North African”. The sample size was determined based on the number of pre-specialty students willing to participate in the reflection after being eye-tracked.

### Procedure and measures

#### Simulation

Vanderbilt nursing students train in a simulated hospital room containing standard medical equipment and a SimMan3G advanced patient simulator manikin from Laerdal Healthcare. The simulated environment is designed to replicate a real hospital setting, complete with medical supplies such as IV stands, medication carts, diagnostic tools, and monitoring devices to provide a realistic and immersive training experience. The primary goal of nursing simulations is to enhance students’ clinical decision-making, patient assessment, and procedural skills in a controlled environment where students can safely practice and refine these competencies. Simulation sessions last 20 min on average, during which students enter the room to perform routine evaluations, such as taking vital signs, assessing patient history, and monitoring symptoms presented by the manikin. Based on their evaluations, students then perform prescribed treatments, which include administering medications, initiating IV fluids, providing oxygen therapy, and documenting their actions. The simulations in this study were part of second-semester prelicensure nursing students’ coursework. These simulations were team-based where students were randomly grouped together in teams of two to three students.

#### Eye-tracking

All students provided their informed consent to collect video and audio data as they performed their training activities, and 65 students volunteered to wear Tobii 3 eye-tracking glasses. Eye-tracked students were fitted and calibrated before entering the simulation room. Eye-tracking glasses were removed upon finishing the simulation scenario. Tobii 3 eye-tracking glasses function at a sampling rate of 50 Hz. Raw gaze coordinates were collected as well as egocentric video and audio footage of the students’ simulation scenarios.

#### Guided reflection

Twenty-five students who were eye-tracked during this simulation returned to the lab and completed the guided reflection, implemented on the Qualtrics survey platform, within 2 weeks of their simulation session. The guided reflection was designed as a complementary activity rather than a replacement of traditional debriefing. Unlike the immediate, semi-structured debrief led by the instructor, which focused on group discussion and general performance takeaways, the guided reflection was an individualized experience that encouraged students to analyze their performance event-by-event with video support. The user experience of the guided reflection was grounded in evidence-based debriefing practices, as will be described in this section. The reflection also allowed us to collect self-report data on students’ performance, cognitive states, and behaviors to determine the degree to which basic eye movements correspond to high-level, domain-specific cognitions.

**Fig. 1:**
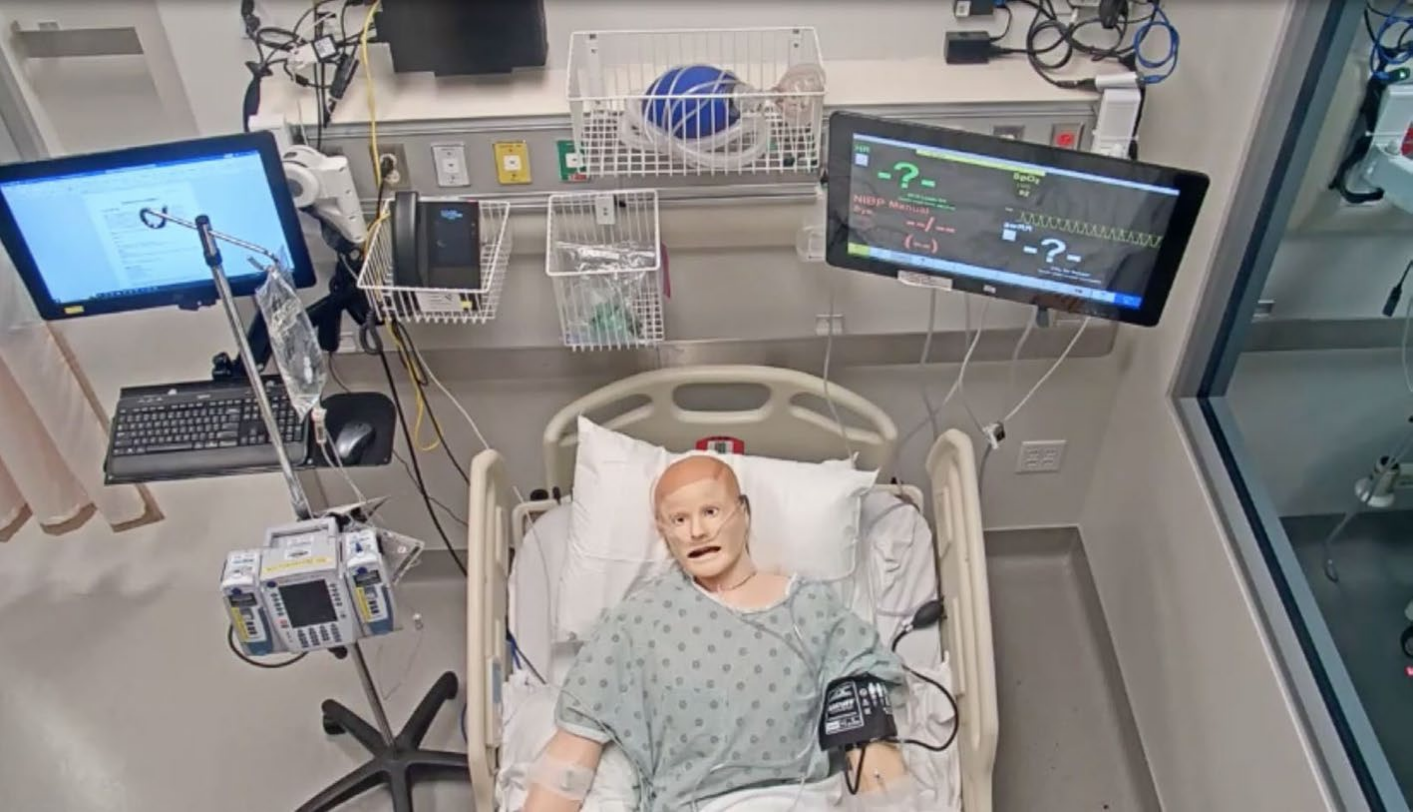
Overhead footage of a simulation room

To begin the guided reflection, students viewed the overhead footage of their simulation session, recorded using a system routinely used by the nursing school at Vanderbilt (Fig. 1). The footage was captured from a ceiling-mounted camera positioned above the simulation patient’s bed, angled to display the patient, the patient history screen, and the vital signs monitor. Students are familiar with this type of footage, as students who are not actively participating in a simulation watch the overhead footage view in real time from a separate room. While this view does not encompass all areas of the room, most notably the medication cabinet, it focuses on the patient and the immediate care environment, ensuring that key assessments and interventions are visible. This footage served as the initial step in the reflection process, as it provided students with an objective reminder of their simulation performance before engaging in a more detailed and structured reflection.

Immediately following the overhead footage, we asked students to complete a Likert version of the Creighton Competency Scale, a scale for self-assessing demonstrated competency, and an individual performance scale (Hayden et al., 2014; Todd et al., 2008). The individual performance scale contained five items (i.e., “Engagement”, “Confidence”, “Patient Safety”, “Positive Patient Outcomes”, “Scenario Objective Completion”) and students rated themselves between 0 and 10 on each item for a maximum score of 50. Students rated to what degree they learned something new about their simulation from the overhead footage (1- “Not at all”, 5- “A great deal”), and they were asked to elaborate on what they learned. Students also rated their confidence in completing a similar scenario but in the real-world, and they rated their general confidence level toward participating in future simulations. Further detail on the questions can be found in our preregistration (https://osf.io/fwvd6).

**Fig. 2:**
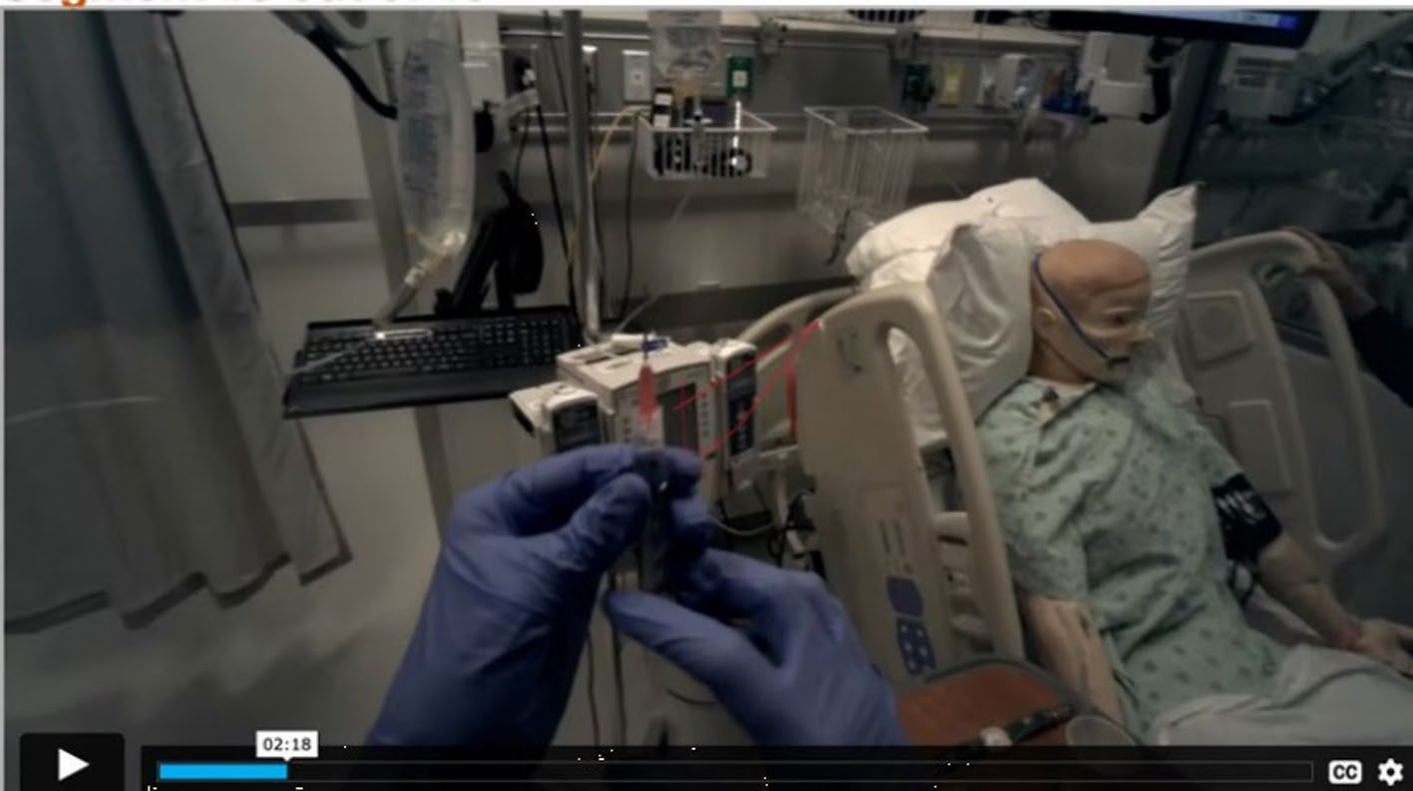
Egocentric eye-tracking footage captured by Tobii Glasses 3. The red circle represents the student’s fixations, and the red lines represent saccades

Next, we presented students with their own egocentric eye-tracking footage from the simulation (Fig. 2). As students watched their eye-tracking footage, we asked them to segment their simulation footage into meaningful event units by pressing a button when they “believe one meaningful event ends and another event begins”. These are standard instructions used to collect behavioral event segmentation data (Zacks, 2020). Prior research has shown that event segmentation is largely consistent across first-person and third-person videos (all depicting other people’s activities), suggesting that segmentation is driven primarily by the structure of the activity rather than low-level visual features (Swallow et al., 2018). Additionally, research on gaze reinstatement suggests that viewing one’s own gaze patterns during retrieval can facilitate memory by reactivating encoding-related cognitive processes (Wynn, et al., 2022). This research implies that presenting students with their gaze data may help them recall what was happening and what they were thinking at that time, potentially enhancing the accuracy and depth of their reflections. This work supports the validity of using first-person footage to examine event segmentation in applied learning contexts such as clinical training.

**Fig. 3:**
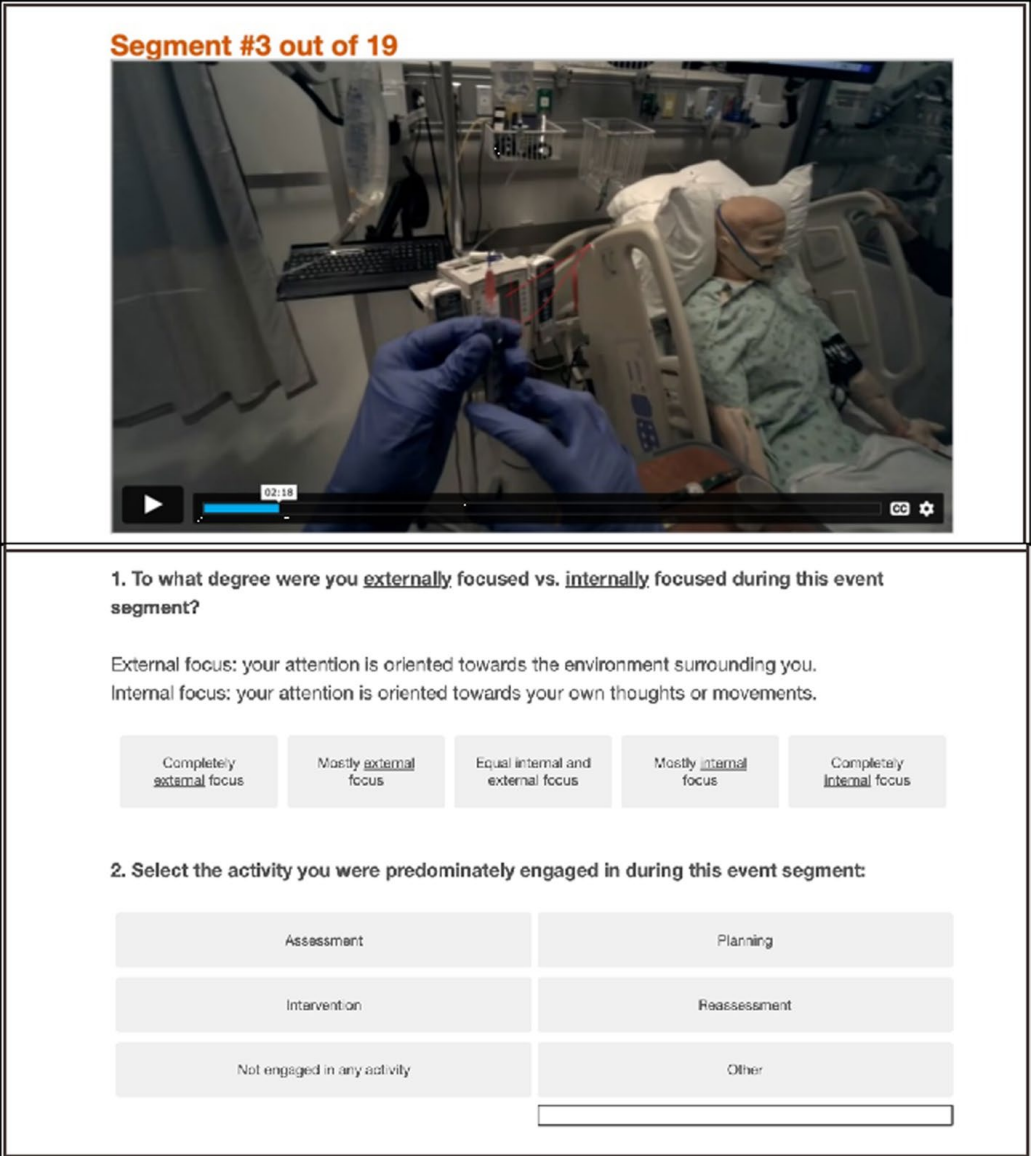
Student’s view of their event-level guided reflection questions

After students segmented their entire simulation into distinct event units, each identified event was shown as a looping video clip, one at a time. Before proceeding to the next event, students answered ten questions designed to induce reflection and characterization of their experience during that event. These questions were grounded in evidence-based best practices in debriefing (Decker et al., 2021). First, students were asked to select what their primary goal was during the given event. The options included each step of the nursing process (“Assessment”, “Planning”, “Intervention”, “Reassessment”; Newton et al., 2019) as well as “No primary goal” and “Other”. Second, students responded to what degree they felt they achieved their primary goal on a 5-point Likert scale from “Strong disagree” to “Strongly agree”. Third, students were given a list of 20 possible actions they might have engaged in during their event. Students were asked to categorize items into three possible bins (“Actions you took to achieve the primary goal of this segment”, “Actions you SHOULD have taken to achieve the primary goal of this segment, but did not take”, “Actions you should NOT have taken during this segment, but did take”). Fourth, students reported the degree to which they were internally versus externally focused on a 5-point Likert scale of “Completely external” to “Completely internal”. Students also reported the degree to which they were working individually versus as a team during that event, and then they rated the quality of teamwork exhibited during that event on a 5-point Likert scale from “Unacceptable” to “Perfect”. For question seven and eight, students rated the effectiveness of their communication with the patient and with the team, respectively. Each question included a 3-point Likert scale where they rated their communication as “Less than necessary”, “As much as necessary”, or “More than necessary”. Finally, students reflected on their self-efficacy by reporting the degree to which they felt confident in their abilities during that event segment on a 5-point Likert scale. (Fig. 3).

To finish the guided reflection, students completed a post-reflection version of the Creighton Competency scale, individual performance scale, and confidence questions. Additionally, students rated to what degree they learned something new about their simulation from the reflection (1- “Not at all”, 5- “A great deal”), and they were asked to elaborate on what they learned. These measures were collected once again in order to make pre- and post-reflection comparisons. Students also completed a version of the Debriefing Assessment for Simulation in Healthcare (DASH) scale to evaluate the quality of the guided reflection as a form of debriefing.

## Results

This study focuses on the relationship between performance judgments and gaze behavior within participant-defined events, leveraging students’ event segmentation structure. While multiple measures were collected during the guided self-reflection phase, including some administered multiple times, only those relevant to individual events are analyzed in this paper. As a result, several pre-registered hypotheses related to self-rated confidence, competency, performance, and DASH scores are not included in these analyses. These measures are the focus of a different manuscript that evaluates the effectiveness of the guided reflection system as a teaching intervention. That manuscript examines the system’s impact on student memory, metacognitive judgment accuracy, and follow-up performance. Because those analyses extend beyond the scope of this study, they are not reported here but will be submitted separately as a more education technology-focused research and development study.

### Descriptive statistics

#### Eye-tracking and event segmentation

The mean duration of eye-tracking simulation footage was 20.4 min (*SD* = 9.8 min). The mean number of events a participant segmented their simulation into was 13 (*SD* = 5.1) and the mean event duration was 1 min and 52 s (*SD* = 2 min and 18 s). The 25 participants segmented a total of 323 events. 11 events were excluded because participants reporting them as accidental segmentations, so 312 segments were used in analyses.

XY coordinates were transformed into gaze metrics using the Tobii Pro Lab’s built-in I-VT algorithm (Tobii Technology, 2012). Saccades were defined as changes in recorded fixation position that exceeded 0.2° with either a velocity that exceeded 30°/s or an acceleration that exceeded 9,500°/s2. From these, we computed the mean fixation duration and mean saccade amplitude by averaging across fixations and saccades in student-marked event. Mean fixation duration (*M* = 388.24, *SD* = 135.79), mean saccade amplitude (*M* = 8.64, *SD* = 1.93), and mean pupil diameter (*M* = 4.52, *SD* = 0.47) were extracted at the event-level.

### Multilevel modeling of gaze as a predictor of performance judgments

We employed multilevel modeling to handle the nested structure of our data, with Level 1 as the event (each participant-defined segment) and Level 2 as the participant (Hox et al., 2017). This approach accounts for the fact that multiple observations (events) are clustered within each person. We conducted our analyses in R using the lme4 package (Bates et al., 2015).

We ran four pre-registered multilevel models using a tear-down strategy to find the model of best fit. Each of 4 planned models tested the impact of 8 predictor variables on one key event-level outcome: Rated quality of teamwork for each event, rated achievement of the primary goal for the event, effective communication during the event (average of ratings for communication with patient and with team), and confidence in abilities during the event.

The predictor variables include three basic eye movement parameters: Mean fixation duration during the event, mean saccade amplitude during the event, and mean pupil diameter during the event. In addition, four task variables were included as predictors: the number of different manual actions identified during the event, the number of different communication events, the number of reading/writing actions, and whether the event reflects a new task. Finally, degree of internal focus was included in each model as a covariate.

We began with maximal models that included random intercepts for participants and random slopes for our within-subject predictors (e.g., fixation duration, pupil diameter, saccade amplitude). To identify the optimal random effects structure, we used a stepwise "tear-down" approach: each predictor was tested by comparing a model with both a fixed effect and a random slope to a model with only a fixed effect, using AIC and BIC to evaluate model fit. Random slopes that did not improve model fit were removed.

Once the random effects structure was finalized, we applied a similar tear-down approach to the fixed effects. Starting with the full model, we removed the least significant fixed effect one at a time, stopping once further removals did not improve model fit. This process resulted in simplified models that retained key predictors while balancing parsimony and fit.

In the final models, all predictors remained fixed effects only, meaning we estimated one effect across all participants, rather than random slopes, which would allow effects to vary across individuals. Below we present key findings from the four models of best fit (Table 1) but see our OSF for more information on these analyses and Appendix 1 for detailed results from each step of the tear-down procedure, as well as model comparisons. The values presented in Table 1 are unstandardized betas.

**Table 1:** Gaze-Outcome Model Summary Results

Predictor	Model 1: Teamwork Quality	Model 2: Goal Achievement	Model 3: Communication	Model 4: Self-efficacy
Fixation duration	.001*			
Saccade amplitude				.06*
Pupil diameter	.32**		.20*	
Manual actions				
Communications	.12*		.13**	
Reading/writing				
New task (1 = new)				
Internal focus		-.20***		-.15*

^*^p < .05, **p < .01, **p < .001

#### Model 1—team quality as an outcome

Participants completed the 5-point Likert scale on teamwork quality only when relevant, that is, if they were working within a team. In 45 events, students indicated they were not involved in team-based activities. This resulted in 268 events where students rated their team quality, ranging from ‘unacceptable’ to ‘perfect.’

**Fig. 4:**
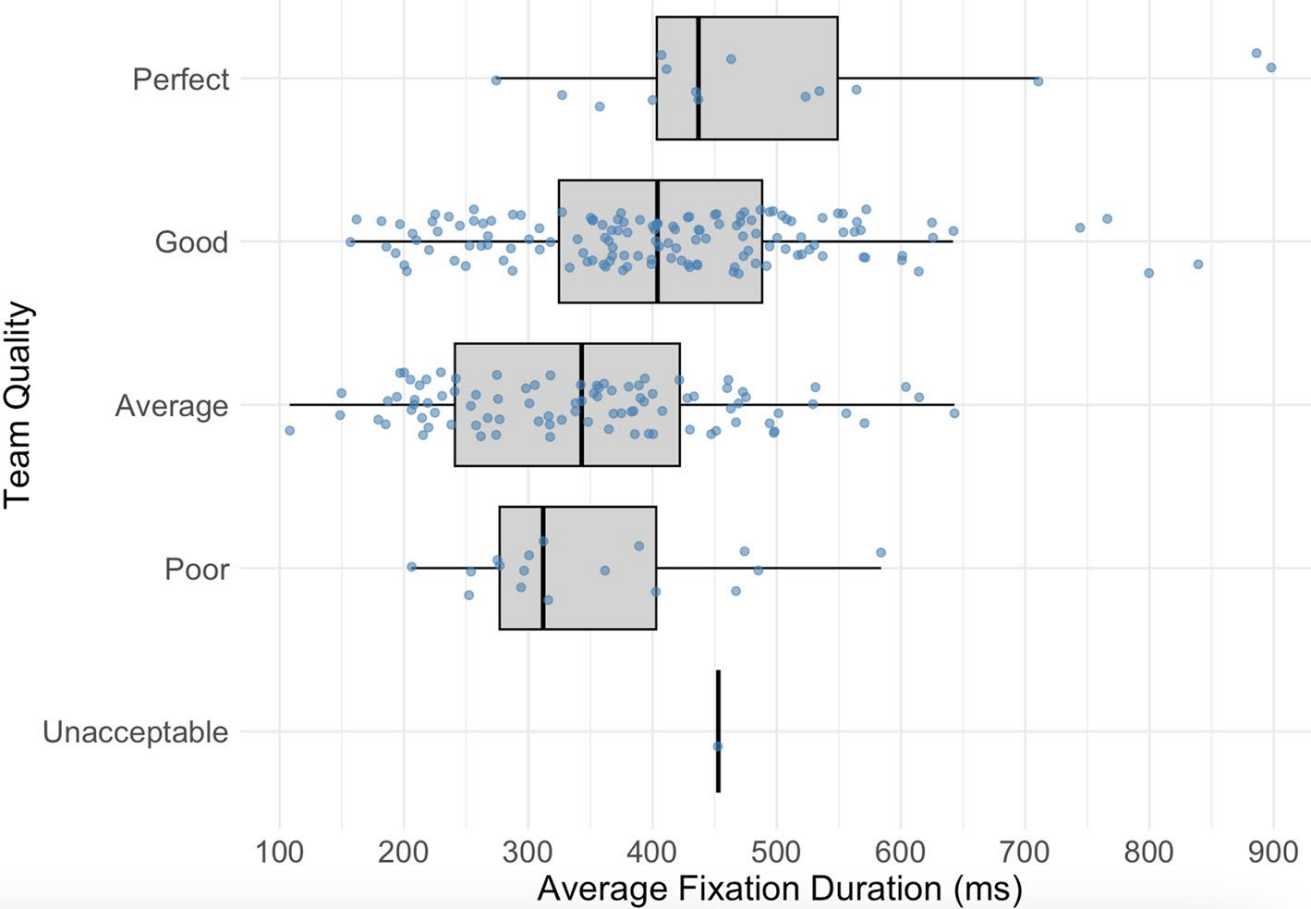
The relationship between mean fixation duration and team quality

Of the 8 predictors, mean fixation duration, mean pupil diameter, and number of communication actions were the final predictors in the best fitting model. Each predictor was positively associated with teamwork quality (Mean fixation duration: *b* = 0.001 *t*(267) = 2.57, *p* = 0.011; Mean pupil diameter: *b* = 0.32 *t*(158) = 2.82, *p* = 0.005; Communication actions: *b* = 0.12 *t*(263) = 2.53, *p* = 0.012). The model accounted for approximately 28% of variance in achievement (Marginal *R*^*2*^ = 0.280). See, for example, the relationship between mean fixation duration and team quality in Fig. 4.

#### Model 2—primary goal achievement as outcome

Of the 8 predictors, internal focus was the only predictor that remained in the best fitting model when clustering by student. Increased internal focus was negatively associated with achievement scores (*b* = -0.20 *t*(306) = − 3.59, *p* < 0.001). The model accounted for approximately 14% of variance in achievement (Marginal *R*^*2*^ = 0.144).

#### Model 3—effective communication as outcome

For questions regarding patient and team communication, students rated their communication as ‘More than necessary,’ ‘Less than necessary,’ or ‘As much as necessary.’ These ratings were coded as 1, − 1, and 0, respectively. To derive a measure of effective communication, students’ scores for both questions were summed, creating a composite outcome variable. Among the eight predictors examined, mean pupil diameter and the number of communication actions emerged as the strongest predictors in the best-fitting model for communication scores. These predictors were positively associated with effective communication (Mean pupil diameter: *b* = 0.20 *t*(196) = 2.08, *p* = 0.039; Communication actions: *b* = 0.13 *t*(304) = 2.97, *p* = 0.0033). The model accounted for approximately 26% of variance in achievement (Marginal *R*^*2*^ = 0.263).

#### Model 4—self-efficacy as outcome

**Fig. 5:**
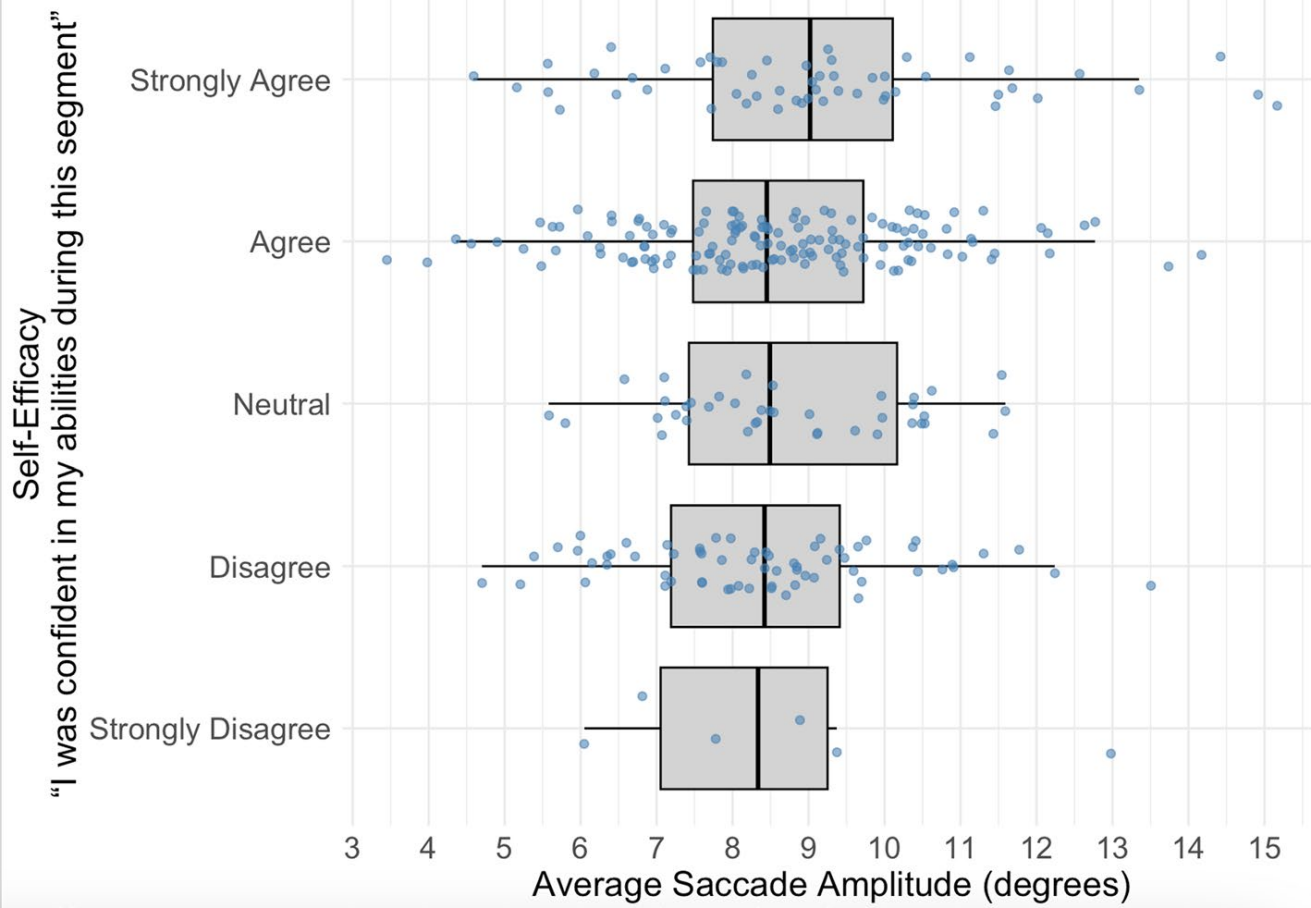
The relationship between mean saccade amplitude and self-efficacy scores

Of the 8 predictors, internal focus and mean saccade amplitude were the remaining predictors in the best fitting model for self-efficacy scores. Similar to achievement in Model 2, increased internal focus was negatively associated with self-efficacy scores (*b* = -0.15 *t*(303) =  − 2.16, *p* = 0.031). Shown in Fig. 5, mean saccade amplitude was positively associated with self-efficacy scores (*b* = 0.06 *t*(235) = 2.01, *p* = 0.045). The model accounted for approximately 16% of variance in achievement (Marginal *R*^*2*^ = 0.162).

#### Focus follow-up

To determine whether the strong impact of internal focus on goal achievement and self-efficacy is a sign of an indirect path from gaze to outcome via focus, we analyzed internal focus as a dependent variable. There were no significant relationships between gaze and internal focus present (Table 2).

**Table 2:** Output of multilevel model with internal focus as dependent variable

Term	Estimate	St. Error	Statistic	*p*-value
Intercept	3.50	0.689	5.08	< .001
Mean fixation duration	− 2.379e-04	2.755e-04	− 0.864	0.388
Mean saccade amplitude	− 0.041	0.024	− 1.726	0.086
Mean pupil diameter	− 0.075	0.138	-0.542	0.589

### Investigating the homogeneity of gaze-performance judgment associations

#### Gaze as predictors across event types

The objective of this study was to examine gaze predictors within participant-defined events, rather than across an entire timeline that encompasses heterogeneous events. The dynamic nature of the simulation learning environment introduces substantial variability, both within and across participants. To account for potential differences in gaze-performance relationships across different stages of the nursing process, we leveraged students’ self-reported primary goals to define event types. During the reflection, students identified the primary goal of each event they segmented, selecting from predefined categories aligned with the nursing process: Assessment, Planning, Intervention, Reassessment, Other, or No goal. These self-reported goals were used as indicators of event type in our analyses.

**Fig. 6:**
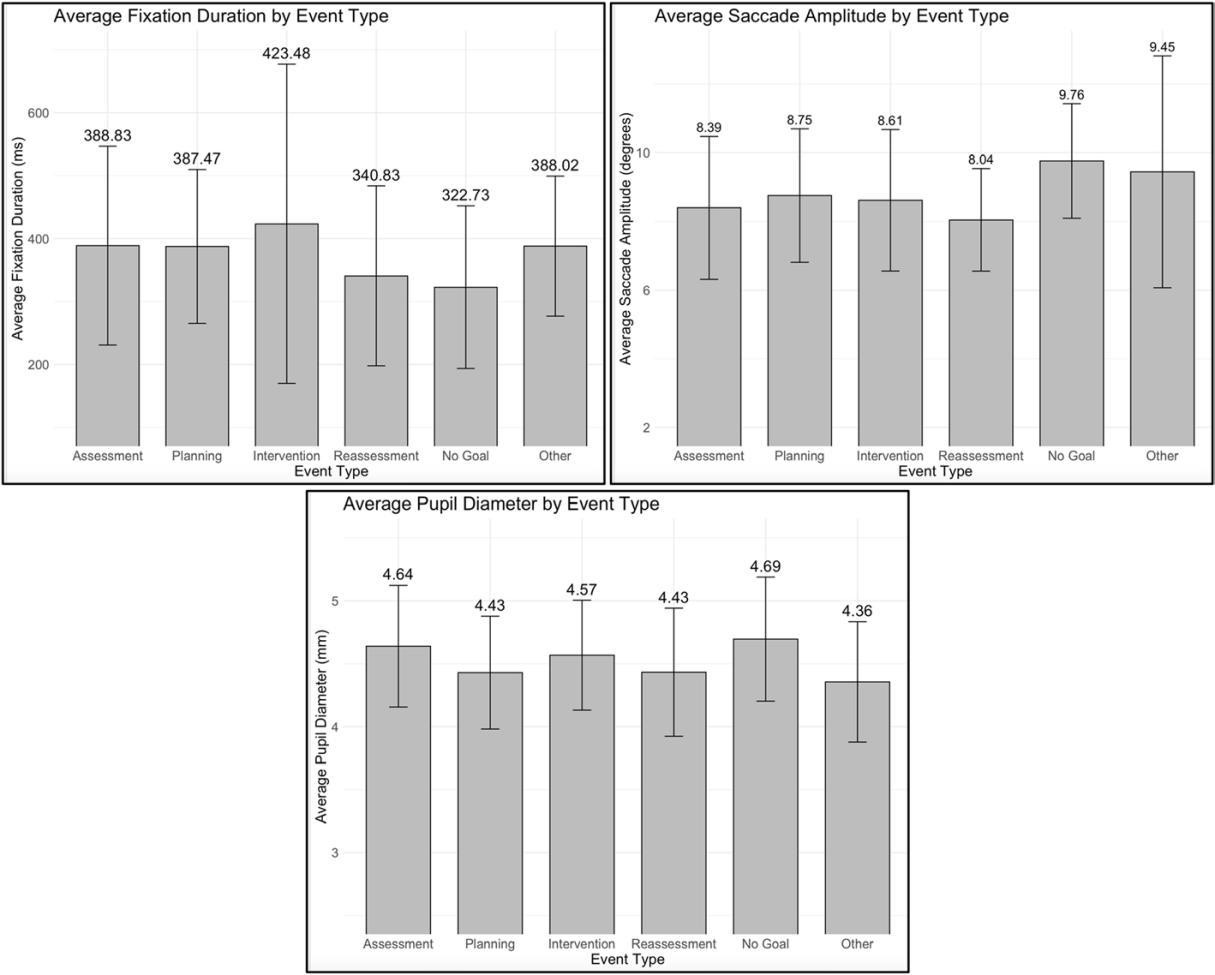
Visualization of gaze parameters categorized by event types. Error bars are standard deviations

Although not pre-registered, we reran each best-fitting model incorporating event type as an additional predictor to assess the generalizability of our findings across these categories. This allowed us to evaluate potential interactions between event type and the strength of gaze predictors on performance judgments. In total, students labeled 54 events as “Assessment, 51 as “Planning”, 120 as “Intervention”, 24 as “Reassessment”, 52 as “Other”, and 11 as “No Goal”. The sub-plots in Fig. 6 show the variance in gaze parameters across event type.

Our follow-up analyses did not reveal a significant interaction between event type and gaze predictors in relation to performance judgments. However, two interactions approached significance at the *p* < 0.10 level. The first was between mean fixation duration and the event type “Reassessment” on teamwork quality (*b* = 0.004, *p* = 0.077), indicating that the relationship between fixation duration and teamwork quality was stronger or more positively associated during Reassessment than during the reference event, No goal. The second interaction involved mean pupil diameter and the event type “Intervention” on teamwork quality (*b* = 0.796, *p* = 0.092), suggesting a stronger positive association between pupil diameter and teamwork quality during Intervention than during No goal. While these interactions did not reach the conventional threshold for statistical significance (*p* < 0.05), they may suggest trends worthy of further exploration. Additionally, no significant differences were found in gaze parameters across the event types. These results suggest there remains a degree of consistency in gaze-performance relationships across stages of the nursing process. See Appendix 2 for detailed analyses.

#### Examining gaze patterns within and across events

Participant-defined events serve as the unit of analysis in this study. To determine whether these events meaningfully capture shifts in cognitive or attentional states and evaluate the temporal dynamics of gaze behavior within and around participant-defined events, we conducted three complementary analyses. We first compared gaze patterns across broader portions (beginning, middle, end) of each event to test for variations in gaze parameters as participants generally develop initial goals, implement actions, and complete goals. We then zoomed in on finer time windows, specifically the first 5 s versus the second 5 s of an event, to test for processing patterns (e.g., ambient-to-focal shift) very early in events observed by Eisenberg and Zacks (2016). Finally, we examined gaze changes across event boundaries in two time windows (last 1 or 5 s of the prior event vs. first 1 or 5 s of the current one), to assess whether transitions marked shifts in attention or cognitive load.

**Consistency of gaze patterns within an event.** Given the dynamic nature of nursing simulations, where multiple actions and decisions often occur within a short time frame, it would be reasonable to expect different gaze patterns between the beginning, middle, and end of these events. To further explore the appropriateness and utility of analyzing gaze metrics and perceived task outcomes at the level of participant-defined events, we examined whether significant differences existed in gaze behavior (fixation duration, saccade amplitude, pupil diameter) at different phases of an event. Specifically, we divided each event into three equal parts—beginning, middle, and end—based on its total duration. By analyzing gaze patterns within these event parts and clustering the data by participant, we aimed to identify potential variations in attention and scene searching over the course of the event. However, our results showed no significant differences in mean fixation duration, saccade amplitude, or pupil diameter between the different event phases, indicating consistency in gaze behavior throughout participant-defined events. Descriptive statistics can be found in Table 3 and multilevel models can be found in Appendix 2.

**Table 3:** Comparison of gaze metrics between beginning, middle, end of events

	Mean Fixation Duration (ms)	Mean Saccade Amplitude	Mean Pupil Diameter (mm)
Beginning of Event	393(*SD* = 185)	8.83(*SD* = 2.91)	4.49(*SD* = 0.48)
Middle of Event	380(*SD* = 195)	8.66(*SD* = 2.75)	4.54(*SD* = 0.50)
End of Event	401(*SD* = 181)	8.71(*SD* = 2.48)	4.51(*SD* = 0.51)

As mentioned previously, Eisenberg and Zacks (2016) investigated gaze patterns in relation to event structure when viewing naturalistic activity and concluded that just as is known with static images (Pannasch et al., 2008; Unema et al., 2005), in dynamic scene viewing there is an initial ambient processing phase at the start of participant-defined events where saccade amplitude is high and fixation duration is low and following this phase is a focal viewing phase where saccade amplitude is low and fixation duration is high. As an extension of the work of Eisenberg and Zacks (2016), we evaluated whether our stimulus show a similar pattern. We found no significant difference between the first five seconds and second five seconds of an event (fixation duration: *b* = − 33.28, *t*(611) = − 1.34, *p* = 0.182; saccade amplitude: *b* = -0.464, *t*(617) = − 1.70, *p* = 0.09; pupil diameter: *b* = − 0.00, *t*(611) = − 0.123, *p* = 0.902). Table 4 presents the means and standard deviations of these comparisons.

**Table 4:** Comparison of Gaze Metrics between 1st and 2nd 5 Seconds of an Event

	Mean Fixation Duration	Mean Saccade Amplitude	Mean Pupil Diameter (mm)
1st 5 s of Event	412(*SD* = 378)	8.97(*SD* = 3.92)	4.54(*SD* = 0.52)
2nd 5 s of Event	377(*SD* = 269)	8.50(*SD* = 3.45)	4.55(*SD* = 0.53)

**Gaze pattern change across an event boundary.** If there is a degree of homogeneity across event types as well as within events, the necessity of evaluating gaze and perceived task outcomes specifically at participant-defined events comes into question. This raises a key consideration: Are participant-defined events meaningful in terms of capturing distinct cognitive or attentional shifts? Although not part of our pre-registered analysis, we sought to determine whether significant changes in gaze patterns could be observed as participants transitioned through the boundaries of these events.

Our analysis revealed that mean fixation duration was significantly greater during the first 5 s of an event (*M* = 412, *SD* = 378) compared to the last 5 s of the prior event (*M* = 348, *SD* = 271; *b* = − 63.38, *t*(624) = − 2.58, *p* = 0.0101), suggesting that participants may allocate more attentional resources at the onset of an event, possibly due to the need to process new information or establish task goals. In contrast, there were no significant differences in fixation behavior between the first (*M* = 237, *SD* = 166) and last 1 s of an event (*M* = 227, *SD* = 158; *b* = − 9.96, *t*(433) = − 0.652, *p* = 0.515), indicating that gaze dynamics may not fluctuate as sharply within such short time windows.

Additionally, neither saccade amplitude nor pupil diameter varied significantly between event boundaries when comparing the first and last five seconds (saccade amplitude: *b* = − 0.05, *t*(623) = -0.185, *p* = 0.853 (${M}_{\text{First Five}}$ = 8.97, ${SD}_{\text{First Five}}$ = 3.92; ${M}_{\text{Last Five}}$ = 8.92, ${SD}_{\text{Last Five}}$ = 3.30); pupil diameter: *b* = − 0.006, *t*(624) = -0.242, *p* = 0.809 (${M}_{\text{First Five}}$ = 4.54, ${SD}_{\text{First Five}}$  = 0.524; ${M}_{\text{Last Five}}$ = 4.54, ${SD}_{\text{Last Five}}$ = 0.525)) and when comparing the first and last second (saccade amplitude: *b* = 0.107, *t*(456) = 0.219, *p* = 0.827 (${M}_{\text{First Sec}}$ = 8.96, ${SD}_{\text{First Sec}}$  = 5.15; ${M}_{\text{Last Sec}}$ = 9.05, ${SD}_{\text{Last Sec}}$ = 5.71); pupil diameter: *b* = -0.03, *t*(423) = − 0.854, *p* = 0.394 (${M}_{\text{First Sec}}$ = 4.58, ${SD}_{\text{First Sec}}$  = 0.535; ${M}_{\text{Last Sec}}$ = 4.57, ${SD}_{\text{Last Sec}}$ = 0.520)). This lack of change in saccade amplitude suggests that the spatial breadth of visual exploration remained relatively stable across events, while the absence of significant pupil diameter fluctuations indicates that cognitive load may not have differed dramatically between event boundaries.

## General discussion

In this study, we uncovered several reliable relationships between gaze behavior and event-level performance judgments based on pre-registered analyses. Increased fixation duration and pupil diameter predicted higher student-rated teamwork quality, while increased pupil diameter predicted effective communication. Further, we found mean saccade amplitude was positively associated with self-efficacy which aligns with prior related work (Vatral et al., 2023). In follow-up analyses that were not pre-registered, we investigated the homogeneity of these gaze to performance judgment associations. We did not detect significant variation in these relationships across event types, and gaze parameters did not differ significantly between the beginning, middle, and end of events. However, there was a significant increase in fixation duration during the first five seconds of an event compared to the last five seconds of the previous event, suggesting an initial encoding phase at an event boundary. The absence of a significant difference in gaze parameters between the one second before and one second after an event boundary aligns with recent event perception literature suggesting that there is an event-integration window allowing for flexible information integration within a ± 3 s range (Lee & Levin, 2024). The lack of a significant difference in gaze parameters in the one second before and after an event boundary may reflect the temporal insensitivity of behavioral segmentation, as seen in normative event segmentation research (Sasmita & Swallow, 2023), or it may indicate that eye movements align with an event-integration window, with gaze shifts emerging only after this window threshold. We also found increased internal focus significantly predicted decreased achievement and self-efficacy scores at the event level, supporting research in nursing education (Benner, 1984; Manetti, 2019). These results do suggest that even in a dynamic and context-dependent environment, there are reliable links between gaze, cognition, and performance outcomes. This contribution aligns with the eye-mind link (Just & Carpenter, 1980), which posits that eye movements provide insight into underlying cognitive processes. The observed relationships between fixation duration, pupil diameter, and event-level judgments suggest that gaze behavior reflects cognitive engagement and processing demands during clinical decision-making.

In our analysis of intra- and inter-event structure, we found that mean fixation duration was significantly greater during the first five seconds of an event compared to the last five seconds of the previous event. Diverging from work of Eisenberg and Zacks (2016), we did not find a significant difference in fixation duration or saccade amplitude from the first five seconds of an event compared to the second five seconds of an event. Eisenberg and Zacks (2016) found more ambient processing takes place at an event boundary (decreased fixation duration, increased saccade amplitude) followed by more focal processing (increased fixation duration, decreased saccade amplitude). Although their stimuli were also dynamic, these differences could appear due to the distinct differences between the stimuli being segmented. Eisenberg and Zacks (2016) eye-tracked participants while they segmented short clips of an unfamiliar person doing everyday tasks (e.g., cooking, decorating). In this context, event boundaries correspond with more perceptually salient changes such as movement or visibly manifested goals, for example moving from chopping vegetables to decorating a cake. Such changes likely induce more ambient processing in order to understand the scope of what is happening in the new event. In our stimuli, students’ eye-tracking footage reflects first-person experience of a clinical event, and their event boundaries likely reflect more conceptual rather than perceptual changes such as goal shifts within the nursing process or clinical decision-making errors. For instance, the same physical actions can occur (e.g., using a stethoscope) but have a different conceptual significance depending on whether they are part of an initial assessment or a follow-up assessment. An initial focused assessment and a follow-up assessment can present the same movements or objects but hold very different clinical meanings. At that start of a new event in a clinical scenario, it could be less useful to engage in ambient processing because the student likely has a thorough understanding of the visual scene and the event at a global level but instead needs to engage in focal processing to make sense of what conceptual shift took place.

Before concluding, it is important to consider one potential issue with our analysis. We assessed the impact of gaze parameters on participant ratings of performance in a situation where those participants had actually viewed their gaze records. This affords the possibility that at least some of the relationships we observed may be complicated by participant inferences about their performance that were derived from their observations of their gaze instead of the direct impact of gaze on experiences. We consider this issue relatively minor, however, for two reasons. First, one of the parameters, pupil diameter, was not, in fact, available in the replays and gaze-cognition relationships were nonetheless still observed. Second, the actual effect sizes were likely too subtle for participants to be aware of. The difference between the longest and shortest fixation durations across the different levels of teamwork quality were about 100 ms, a parameter that is likely quite subtle to perceive. Finally, any video that includes looking behavior will include perceptual information specifying both gaze shifts (in the form of head movements) and fixation durations (in the form of visibly extended looks), so this issue is likely to be quite general in real-world gaze-recording settings.

That said, it will be useful in future research both to assess gaze-cognition links in a more controlled setting, and to test for links between gaze behavior and more objective measures of performance. In the current project, we were constrained not to interfere with the simulation process, so we could not collect objective ratings from knowledgeable instructors. In addition, for this initial project we wanted to focus on how gaze parameters might be related to variations in student experience that are clearly central to the simulation process. As reviewed above, a key learning goal for these simulations is student self-efficacy (Cabrera-Aguilar et al., 2023; Lee & Ko, 2010) so gaze parameters that may predict these experiences have clear value. For example, if decreased saccade amplitude can be used as part of a model that could detect moments of doubt, or decreased pupil diameter can be used to detect moments where students perceive poor teamwork, then these may be used to guide student to moments where they might employ self-regulation strategies (Brydges & Butler, 2012) to adjust their actions. By leveraging the eye-mind link (Just & Carpenter, 1980), simulation-based learning environments could integrate gaze tracking as a feedback tool to assess attention, cognitive workload, or uncertainty in decision-making, providing instructors with objective insights into students’ thought processes. It is particularly interesting to note that these dynamic cues could be useful both as guides to the post-action debriefing processes that are central to current simulation learning practice (Lee et al., 2020) or even as on-line interventions that might be applied in future simulations while students are in-situ.

We propose that aggregating gaze parameters across participant-defined events is a powerful tool to increase the power of analyses linking gaze and cognition. Grounding analyses by first-person event segmentations ensures interpretability and considers person-level cognition. Previous event segmentation research typically uses generic stimuli, such as short clips of a person cleaning their house, to evaluate perceptual processes (Eisenberg & Zacks, 2016). This prior work has shown that changes in dimensions such as relevant changes in time, location, characters, causality, intention most frequently elicit the perception of event boundaries (Radvansky & Zacks, 2014). Working event models contain updated information on each of these dimensions, so it is assumed that as the number of dimension changes increase, the likelihood of perceiving an event boundary also increases (Huff et al., 2014). Our work presents the possibility that there are more internal aspects of cognition that could influence the perception of an event boundary (e.g., conceptual shifts). This takeaway is consistent with recent theoretical efforts to incorporate internal state changes as determinants of event segmentation (Wang et al., 2024). To improve on our analytical approach, it is important to empirically evaluate what predicts event segmentation in hands-on learning experiences and these types of first-person experiences generally. Nonetheless, our work expands on what has been done in eye-tracking research, using event segmentation as an analytical approach, and in event segmentation research, using meaningful and personalized stimuli.

## Data Availability

The dataset collected and analyzed during the current study as well as pre-registered analyses are available in the OSF repository, https://osf.io/fwvd6.

## Model 1 teamwork quality

### Random slope stepwise comparison

AIC/BIC comparisons for random slopes in predicting team quality

**Table:**

DV	Predictor	AIC Comparison			BIC Comparison		
		AIC (With Slope)	AIC (Base)	$\Delta$ AIC	BIC (With Slope)	BIC (Base)	$\Delta$ BIC
Team_Quality	New task	521.39	523.86	2.47	568.02	563.32	− 4.70
Team_Quality	Focus	524.51	523.86	− 0.64	571.14	563.32	− 7.82
Team_Quality	Manual _action	524.88	523.86	− 1.02	571.52	563.32	− 8.19
Team_Quality	Average_duration_of_whole_fixations	526.41	523.86	− 2.54	573.04	563.32	− 9.72
Team_Quality	Average_amplitude_of_saccades	526.72	523.86	− 2.85	573.35	563.32	− 10.03
Team_Quality	Average_whole_fixation_pupil_diameter	527.22	523.86	− 3.36	573.86	563.32	− 10.53
Team_Quality	Communication_action	527.32	523.86	− 3.46	573.96	563.32	− 10.64
Team_Quality	Read_write_action	527.46	523.86	− 3.60	574.09	563.32	− 10.77

The base model is a random intercept only model with all predictors as fixed effects and the “with slope” model is the same model only adding a random slope to the corresponding predictor. When comparing both the AIC/BIC between the base and slope model, the base model leads to more parsimony in all cases.

### Full fixed effects model

Table A: Fixed effects summary for teamwork quality model

**Table:**

	estimate	std. error	statistic	*p* value
(Intercept)	1.957	0.599	3.265	0.001**
Average_duration_of_whole_fixations	0.001	0.000	2.380	0.018*
Average_amplitude_of_saccades	− 0.003	0.020	− 0.153	0.878
Average_whole_fixation_pupildiameter	0.317	0.113	2.813	0.006**
Focus	− 0.041	0.047	− 0.870	0.385
ManualAction	0.030	0.083	0.356	0.722
Communication Action	0.120	0.048	2.497	0.013*
Read/Write Action	0.087	0.087	1.010	0.313
New Task	− 0.122	0.080	− 1.527	0.128

AIC = 523.86, BIC = 563.32.

### Tear-down strategy

1. First, remove the least significant predictor, saccade amplitude.
  Table B: Fixed Effects Summary for Teamwork Quality Model.
  **Table:**
  Term	Estimate	std.error	Statistics	*p* value
  (Intercept)	1.924	0.562	3.425	0.001***
  Average_duration_of_whole_fixations	0.001	0.000	2.441	0.015*
  Average_whole_fixation_pupil_diameter	0.317	0.113	2.814	0.006**
  Focus	− 0.040	0.047	− 0.855	0.393
  Manual Action	0.031	0.083	0.379	0.705
  Communication Action	0.120	0.048	2.495	0.013*
  Read Write Action	0.039	0.086	1.025	0.306
  New Task	− 0.121	0.080	− 1.524	0.129
  AIC = 521.88, BIC = 557.76.
2. Model fit improved with removal of saccade amplitude. Next, remove manual action.
  Table C: Fixed effects summary for teamwork quality model
  **Table:**
  Term	Estimate	Std.error	statistic	*p* value
  (Intercept)	1.929	0.562	3.434	0.001***
  Average_duration_of_whole_fixations	0.001	0.000	2.465	0.014*
  Average_whole_fixation_pupil_diameter	0.321	0.112	2.853	0.005**
  Focus	-0.039	0.047	−6.840	0.401
  Communication Action	0.120	0.048	2.493	0.013*
  Read/Write Action	0.088	0.086	1.015	0.311
  New Task	-0.125	0.079	-1.591	0.113
  AIC = 520.03, BIC = 552.31.
3. Model fit improved with removal of manual action. Next, remove focus.
  Table D: Fixed effects summary for teamwork quality model.
  **Table:**
  	Estimate	std, error	statistic	*p* value
  (Intercept)	1.794	0.538	3.332	0.001**
  Average_duration_of_whole_fixations	0.001	0.000	2.499	0.013*
  Average_whole_fixation_pupil_diameter	0.325	0.112	2.901	0.004**
  Communication Action	0.124	0.048	2.576	0.011*
  Read/Write Action	0.086	0.086	0.994	0.321
  New Task	− 0.124	0.079	− 1.569	0.118
  AIC = 518.72, BIC = 547.42.
4. Model fit improved with removal of focus. Next, remove read/write actions.
  Table E: Fixed Effects Summary for Teamwork Quality Model
  **Table:**
  	Estimate	Std, error	Statistic	*p* value
  (Intercept)	1.917	0.526	3.642	0***
  Average_duration_of_whole_fixations	0.001	0.000	2.521	0.012*
  Average_whole.fixation_pupil_diameter	0.305	0.111	2.754	0.007**
  Communication Action	0.129	0.048	2.704	0.007**
  New Task	−0.136	0.078	−1.750	0.081
5. Model fit improved with removal of read/write actions. Next, remove new task.
  Table F: Fixed Effects Summary for Teamwork Quality Model
  **Table:**
  term	estimate	std,error	statistic	*p* value
  (Intercept)	1.796	0.528	3.405	0.001***
  Average_duration_of_whole_fixations	0.001	0.000	2.565	0.011*
  Average_whole.fixation_pupil_diameter	0.315	0.112	2.815	0.005**
  Communication, Action	0.121	0.048	2.532	0.012*
  AIC = 518.72, BIC = 540.25.
6. Model fit (BIC) improved with removal of new task. Next, remove communication action.
  Table G: Fixed effects summary for teamwork quality model
  **Table:**
  term	estimate	std. error	statistic	*p* value
  (Intercept)	1.955	0.537	3.640	0***
  Average_duration_of_whole_fixations	0.001	0.000	2.445	0.015*
  Average_whole.fixation_pupil_diameter	0.317	0.114	2.767	0.006**
  AIC = 522.94, BIC = 540.88.

### Final model for teamwork quality

AIC and BIC either worsened or stayed the same when removing communication action. In other words, the fifth model in our tear-down approach is the final model for teamwork quality.

## Model 2 achievement

### Random slope stepwise comparison

AIC/BIC Comparisons for Random Slopes in Predicting Achievement

**Table:**

DV	Predictor	AIC Comparison			BIC Comparison		
		AIC (With Slope)	(Base) AIC	$\Delta$ AIC	BIC (With Slope)	BIC (Base)	$\Delta$ BIC
achievement	Average_whole_fixation_pupil_diameter	774.58	774.05	− 0.53	823.19	815.19	− 8.01
achievement	manual_action	775.13	774.05	− 1.08	823.75	815.19	− 8.56
achievement	communicationl_action	776.55	774.05	− 2.51	825.17	815.19	− 9.99
achievement	readl_writel_action	777.75	774.05	− 3.70	826.36	815.19	− 11.18
achievement	focus	777.79	774.05	− 3.75	826.41	815.19	− 11.22
achievement	newl_task	777.89	774.05	− 3.84	826.51	815.19	− 11.32
achievement	averagel_amplitudel_ofl_saccades	779.30	774.05	− 5.25	827.92	815.19	− 12.73
achievement	averagel_durationl_ofl_wholel_fixations	780.40	774.05	− 6.35	829.02	815.19	− 13.83

The base model is a random intercept only model with all predictors as fixed effects and the “with slope” model is the same model only adding a random slope to the corresponding predictor. When comparing both the AIC/BIC between the base and slope model, the base model leads to more parsimony in all cases.

### Full fixed effects model

Table H: Fixed effects summary for achievement model

**Table:**

term	estimate	std error	statistic	*p* value
(Intercept)	4.382	0.691	6.339	0***
Averagel_durationl_ofl_wholel_fixations	0.000	0.000	0.078	0.938
Averagel_amplitudel_ofl_saccades	0.020	0.022	0.878	0.381
Averagel_whole.fixationl_pupil l_diameter	− 0.041	0.129	− 0.318	0.751
Focus	− 0.194	0.055	− 3.526	0^***^
Manual Action	0.172	0.100	1.722	0.086
Communication Action	− 0.039	0.058	− 0.662	0.508
Read/Write Action	− 0.148	0.106	− 1.390	0.166
New Task	− 0.004	0.096	− 0.045	0.964

AIC = 774.05, BIC = 815.19, p < 0.001 = ***

### Final model for Achievement after implementing a tear-down approach

Table I Fixed effects summary for achievement model

**Table:**

Term	Estimate	std. error	Statistic	*p* value
(Intercept)	4.399	0.166	26.462	0***
Focus	− 0.197	0.055	− 3.587	0***

AIC = 767.79, BIC = 782.76, p *< *0.001 = ***

## Model 3 effective communication

### Random slope stepwise comparison

**Table:**

DV	Predictor	AIC (With	AIC	$\Delta$	BIC (With	BIC	$\Delta$
		Slope)	(Base)	AIC	Slope)	(Base)	BIC
Communication_sum	new_task	572.26	571.59	− 0.67	620.88	612.73	− 8.15
Communication_sum	communication_action	573.25	571.59	− 1.66	621.87	612.73	− 9.14
Communication_sum	manua_action	574.80	571.59	− 3.21	623.42	612.73	− 10.69
Communication_sum	focus	574.92	571.59	− 3.33	623.54	612.73	− 10.81
Communication_sum	average_amplitude_of_saccades	575.24	571.59	− 3.64	623.85	612.73	− 11.12
Communication_sum	read_write_action	575.57	571.59	− 3.97	624.18	612.73	− 11.45
Communication_sum	average_duration_of_whole_fixations	575.68	571.59	− 4.09	624.30	612.73	− 11.57
Communication_sum	average_whole_fixation_pupil_diameter	575.75	571.59	− 4.15	624.36	612.73	− 11.63

The base model is a random intercept only model with all predictors as fixed effects and the “with slope” model is the same model only adding a random slope to the corresponding predictor. When comparing both the AIC/BIC between the base and slope model, the base model leads to more parsimony in all cases.

### Full fixed effects model

Table J: Fixed effects summary for effective communication model

**Table:**

	Estimate	std, error	statistic	*p* value
(Intercept)	− 1.355	0.534	− 2.539	0.012*
Average_duration_of_whole_fixations	0.000	0.000	0.645	0.519
Average_amplitude_of_saccades	0.016	0.017	0.927	0.355
Average_whole.fixation_pupil _diameter	0.200	0.101	1.976	0.05*
Focus	− 0.058	0.040	− 1.456	0.146
Manual Action	0.046	0.072	0.644	0.52
Communication Action	0.121	0.043	2.804	0.005**
Read/Write Action	0.018	0 077	0.236	0.813
New Task	− 0.046	0.069	− 0.661	0.509

AIC = 571.59, BIC = 612.73

### Final model for Achievement after implementing a tear-down approach

Table K: Fixed effects summary for elective communication model

**Table:**

Term	Estimate	std. error	Statistic	*p* value
[Intercept]	− 1.342	0.453	− 2.963	0.003**
Average_whole_fixation_pupil_diameter	0.203	0.098	2.078	0.039*
Communication Action	0.126	0.043	2.966	0.003**

AIC = 564.04, BIC = 582.73.

## Model 4 Self-efficacy

### Random slope stepwise comparison

AIC/BIC Comparisons for Random Slopes in Predicting Self_efficacy

**Table:**

DV	Predictor	AIC Comparison			BIC Comparison		
		AIC (With Slope)	AIC (Base)	$\Delta$ AIC	BIC (With Slope)	BIC (Base)	$\Delta$ BIC
Self_efficacy	Focus	913.32	913.41	0.09	961.93	954.55	− 7.39
Self_efficacy	Manual action	914.41	913.41	− 1.00	963.03	954.55	− 8.48
Self_efficacy	Communication action	915.39	913.41	− 1.98	964.00	954.55	− 9.46
Self_efficacy	Average_whole_fixation_pupi_diameter	915.79	913.41	− 2.38	964.41	954.55	− 9.86
Self_efficacy	Average amplitude of saccades	916.76	913.41	− 3.35	965.38	954.55	− 10.83
Self_efficacy	New task	917.16	913.41	− 3.75	965.77	954.55	− 11.23
Self_efficacy	Read/write action	917.29	913.41	− 3.89	965.91	954.55	− 11.37
Self_efficacy	Average_duration_of_whole_fixations	917.65	913.41	− 4.24	966.26	954.55	− 11.72

The base model is a random intercept only model with all predictors as fixed effects and the “with slope” model is the same model only adding a random slope to the corresponding predictor. When comparing both the AIC/BIC between the base and slope model, the base model leads to more parsimony in all cases.

### Full fixed effects model

**Table:**

Term	Estimate	Std.error	statistic	*p* value
(Intercept)	4.307	0.885	4.869	0***
Average_duration_of_whole_fixations	0.001	0.000	0.046	0.963*
Average_amplitude_of_saccedes	0.059	0.029	2.061	0.04*
Average_whole.fixation_pupil_diameter	−0.221	0.166	−1.332	0.185
Focus	−0.148	0.069	2.147	0.033*
Manual.Action	0.121	0.125	0.971	0.332
Communication Action	0.010	0.074	−0.130	0.896
Read.Write Action	−0.106	0.133	−0.798	0.426
New Task	0.099	0.120	0.823	0.411

AIC = 913.41, BIC = 954.55.

### Final model for Achievement after implementing a tear-down approach

Table M: Fixed Effects Summary for Self-Efficacy Model

**Table:**

Term	Estimate	std.error	statistic	*p* value
(Intercept)	3.450	0.334	10.339	0***
Average_amplitude_of_saccades	0.056	0.028	2.010	0.045*
Focus	− 0.149	0.069	− 2.163	0.031*

AIC = 905.19, BIC = 923.89.

## Gaze as predictors across event types

### There are no significant differences in gaze parameters between any event type

Fixation duration by event type with no goal as reference

**Table:**

Term	Estimate	std.error	statistic	*p* value
(Intercept)	332.348	54.606	6.086	0***
GoalAssessment	46.306	55.592	0.833	0.406
GoalReassessment	36.173	61.475	0.588	0.557
GoalPlanning	62.538	55.938	1.118	0.264
Goallntervention	84.648	53.757	1.575	0.116
GoalOther	43.920	57.780	0.760	0.448

Saccade AMP by event type with no goal as reference

**Table:**

Term	Estimate	std.error	statistic	*p* value
(Intercept)	9.534	0.637	14.966	0***
GoalAssessment	−1.066	0.626	1.701	0.09
GoalReassessment	−1.202	0.693	−1.734	0.084
GoalPlanning	−1.003	0.631	−1.590	0.113
Goallntervention	−1.081	0.607	−1.782	0.076
GoalOther	−0.183	0.653	−0.283	0.777

Pupil diameter by event type with no goal as reference.

**Table:**

Term	Estimate	std.error	Statistic	*p* value
(Intercept)	4.529	0.113	40.015	0***
GoalAssessment	0.008	0.094	0.080	0.936
GoalReassessment	− 0.013	0.104	− 0.120	0.904
GoalPlanning	− 0.092	0.095	− 0.971	0.333
Goallntervention	0.086	0.091	0.938	0.349
GoalOther	− 0.042	0.099	− 0.425	0.671

### There are no significant interactions between event type and gaze predictors in relation to performance judgments

Adding Gase-goal intertractions for best fitting model for teamwork quality

**Table:**

Term	Estimate	Std.error	Statistic	*p* value
(Intercept)	5.614	2.442	2.299	0.022*
Average_duration_of_whole_fixations	−0.002	0.002	−0.867	0.387
GoalAssessment	−3.826	2.607	−1.468	0.143
GoalReassessment	−4.033	2.684	−1.502	0.134
GoalPlanning	−4.015	2.591	−1.549	0.123
Goallntervention	−4.495	2.517	−1.786	0.075
GoalOther	−4.669	2.591	−1.802	0.073
Average_whole.fixation_pupil_diameter	−0.331	0.450	−0.734	0.463
Communication.Action	0.124	0.049	2.556	0.011*
Average_duration_of_whole_fixations:GoalAssessment	0.003	0.002	1.391	0.166
Average_duration_of_whole_fixations:GoalReassessment	0.004	0.002	1.773	0.077
Average_duration_of_whole_fixations:GoalPlanning	0.003	0.002	1.394	0.164
Average_duration_of_whole_fixations:Goallntervention	0.002	0.002	1.052	0.294
Average_duration_of_whole_fixations:GoalOther	0.003	0.002	1.510	0.132
GoalAssessment:Average_whole.fixation_pupil_diameter	0.607	0.482	1,257	0.21
GoalReassessment:Average_whole.fixation_pupil_diameter	0.543	0.510	1.065	0.288
GoalPlanning:Average_whole.fixation_pupil_diameter	0.649	0.486	1.337	0.182
Goallntervention:Average_whole.fixation_pupil_diameter	0.796	0.470	1.694	0.092
GoalOther:Average_whole.fixation_pupil_diameter	0.772	0.493	1.568	0.118

Adding Gaze−Goal Interactions to Best Fitting Model for Achievement

**Table:**

Term	Estimate	Std.error	Statistic	*p* value
(Intercept)	4.214	0.636	6.630	0***
Focus	− 0.242	0.196	− 1.239	0.216
GoalAssessment	− 0.054	0.751	− 0.071	0.943
GoalReassessment	− 0.241	0.883	− 0.273	0.785
GoalPlanning	− 0.013	0.747	− 0.017	0.986
Goallntervention	0.100	0.682	0.147	0.883
GoalOther	0.410	0.714	0.574	0.566
Focus:GoalAssessment	0.249	0.258	0.967	0.334
Focus:GoalReassessment	0.144	0.301	0.480	0.631
Focus:GoalPlanning	0.058	0.239	0.244	0.807
Focus:Goallntervention	0.043	0.212	0.200	0.841
Focus:GoalOther	0.049	0.222	0.222	0.824

Adding Gaze-Goal Interactions to Best Fitting Model for Communication

**Table:**

term		estimate	std.error	statistic	p.value
(Intercept)		− 1.390	0.454	− 3.063	0.002**
Average whole fixation pupil diameter		0.210	0.095	2.222	0.026*
GoalAssessment		0.128	0.489	0.263	0.793
GoalPlanning		0.390	0.482	0.809	0.418
Goallntervention		0.313	0.468	0.669	0.503
GoalReassessment		0.131	0.511	0.256	0.798
GoalOther		0.286	0.492	0.581	0.561
Communication Action		0.132	0.012	11.156	0***
Average_whole.fixation_pupil_diameter:GoalAssessment		− 0.026	0.103	− 0.256	0.798
Average_whole.fixation_pupil_diameter:GoalPlanning		− 0.084	0.102	− 0.821	0.412
Average_whole.fixation_pupil_diameter:Goallntervention		− 0.066	0.099	− 0.671	0.502
Average_whole.fixation_pupil_diameter:GoalReassessment		− 0.026	0.109	− 0.242	0.809
Average_whole.fixation_pupil_diameter:GoalOther		− 0.061	0.105	− 0.581	0.561

Adding Gaze-Goal Interactions to Best Fitting Model for Self-Efficacy

**Table:**

term	Estimate	std.error	statistic	*p* value
(Intercept)	3.589	0.342	10.509	0***
Average amplitude of saccades	0.045	0.035	1.276	0.202
GoalAssessment	− 0.069	0.346	− 0.201	0.841
GoalPlanning	− 0.010	0.346	− 0.028	0.977
Goallntervention	0.085	0.333	0.257	0.798
GoalReassessment	0.011	0.369	0.030	0.976
GoalOther	0.298	0.361	0.827	0.408
Focus	− 0.172	0.018	− 9.536	0***
Average_amplitude_of_saccades:GoalAssessment	0.008	0.038	0.203	0.839
Average_amplitude_of_saccades:GoalPlanning	0.001	0.038	0.023	0.982
Average_amplitude_of_saccades:Goallntervention	− 0.010	0.037	− 0.271	0.786
Average_amplitude_of_saccades:GoalReassessment	− 0.001	0.041	− 0.030	0.976
Average_amplitude_of_saccades:GoalOther	− 0.034	0.039	− 0.853	0.394

## Consistency of gaze patterns within an event

Fixed Effects Summary for Fixation Duration Within-Event Comparisons

**Table:**

Term	Estimate	std.error	statistic	*p* value
Beginning of Event—End of Event	12.565	12.850	0.978	0.591
Beginning of Event—Middle of Event	− 7.526	12.859	− 0.585	0.828
End of Event—Middle of Event	− 20.091	12.902	− 1.557	0.265

Fixed Effects Summary for Saccade Amplitude Within-Event Comparisons

**Table:**

Term	Estimate	std.error	Statistic	*p* value
Beginning of Event—End of Event	0.156	0.197	0.791	0.709
Beginning of Event—Middle of Event	0.118	0.197	0.600	0.820
End of Event—Middle of Event	− 0.038	0.198	− 0.191	0.980

Fixed Effects Summary for Pupil Diameter Within-Event Comparisons

**Table:**

Term	Estimate	std.error	Statistic	*p* value
Beginning of Event—End of Event	− 0.037	0.026	− 1.406	0.338
Beginning of Event—Middle of Event	-0.019	0.026	-0.717	0.753
End of Event—Middle of Event	0.018	0.027	0.685	0.772
